# IAPP - oligomerisation levels in plasma of people with type 2 diabetes

**DOI:** 10.1038/s41598-024-70255-3

**Published:** 2024-08-22

**Authors:** Fabian Rehn, Victoria Kraemer-Schulien, Tuyen Bujnicki, Oliver Bannach, Diethelm Tschoepe, Bernd Stratmann, Dieter Willbold

**Affiliations:** 1 Institut für Physikalische Biologie, Heinrich-Heine-Universität Düsseldorf, Universitätsstr. 1, 40225 Düsseldorf, Germany; 2 Institute of Biological Information Processing (Structural Biochemistry: IBI-7), Forschungszentrum Jülich GmbH, Wilhelm-Johnen-Straße, 52428 Jülich, Germany; 3 attyloid GmbH, Merowingerplatz 1A, 40225 Düsseldorf, Germany; 4 Herz- und Diabeteszentrum Nordrhein-Westfalen, Universitätsklinik der Ruhr-Universität Bochum, Medizinische Fakultät OWL (Universität Bielefeld), Georgstr. 11, 32545 Bad Oeynhausen, Germany; 5 Stiftung DHG (Diabetes I Herz I Gefäße) in der Deutschen Diabetes Stiftung, c/o Deutsches Diabetes-Zentrum (DDZ), Auf´m Hennekamp 65, 40225 Düsseldorf, Germany

**Keywords:** IAPP, Diabetes, Disease driven protein expression, Oligomers, sFIDA, Diabetes complications, Type 2 diabetes, Peptides, Protein folding, Alzheimer's disease

## Abstract

Islet amyloid polypeptide (IAPP) is co-secreted with insulin from pancreatic ß-cells. Its oligomerisation is regarded as disease driving force in type 2 diabetes (T2D) pathology. Up to now, IAPP oligomers have been detected in affected tissues. IAPP oligomer concentrations in blood have not been analysed so far. Using the IAPP single-oligomer-sensitive and monomer-insensitive surface-based fluorescence intensity distribution analysis (sFIDA) technology, levels of IAPP oligomers in blood plasma from healthy controls and people with T2D in different disease stages where determined. Subsequently, the level of IAPP oligomerisation was introduced as the ratio between the IAPP oligomers determined with sFIDA and the total IAPP concentration determined with ELISA. Highest oligomerisation levels were detected in plasma of people with T2D without late complication and without insulin therapy. Their levels stand out significantly from the control group. Healthy controls presented with the lowest oligomerisation levels in plasma. In people with T2D without complications, IAPP oligomerisation levels correlated with disease duration. The results clearly demonstrate that IAPP oligomerisation in insulin-naïve patients correlates with duration of T2D. Although a correlation per se does not identify, which is cause and what is consequence, this result supports the hypothesis that IAPP aggregation is the driving factor of T2D development and progression. The alternative and conventional hypothesis explains development of T2D with increasing insulin resistance causing exhaustion of pancreatic ß-cells due to over-secretion of insulin, and thus IAPP, too, resulting in subsequent IAPP aggregation and fibril deposition in the pancreas. Further experiments and comparative analyses with primary tissues are warranted.

## Introduction

Type 2 diabetes (T2D) is a metabolic disorder that progresses continuously and is characterised by insulin resistance, often in conjunction with impaired insulin secretion. With increasing resistance, β-cells respond with augmented insulin secretion to overcome the resistance (hyperinsulinaemia)^1^. Over time, a decline in insulin secretion is observed as a signal of pancreas exhaustion. One possible cause is the diminishment in β-cell mass, resulting in decreased insulin secretion^2^. Due to the resulting lack of functional regulatory mechanisms for glucose, along with a potentially increasing gluconeogenesis in the liver, this can further aggravate hyperglycaemia, which in turn can favour various diseases, named diabetic late complications^3^. Multiple potential risk factors such as obesity or advancing age^4^, as well as genetic factors contribute to its development^5^. In recent decades, the number of T2D cases, which account for more than 90% of diabetes cases^6^, worldwide has risen considerably, posing an increasingly significant burden on healthcare systems^7^. However, compared to the other types of diabetes, less pronounced hyperglycaemia and decrease in β-cell mass occurs in T2D opening the window for non-insulin based, but ß-cell protective therapies^8,9^.

Alongside the periodically increased insulin production, insulin resistance also elevates the level of IAPP monomer, as both are co-secreted^10^, with a potentially disproportionate increase in the latter^11^. IAPP, initially existing in monomeric state, possesses the potential to aggregate into small soluble oligomers, as well as to accumulate into insoluble fibrils, also called islet amyloid^12^. Due to the decrease in the ß-cell mass, the amount of monomers available for aggregation changes during the course of the disease. In the case of T2D, islet amyloid formation occurs within the islet cells, extracellularly between ß-cells, as well as between ß-cells and endothelial cells^13^. However, experiments with transgenic mice indicate that extracellular amyloid does not induce β-cell apoptosis^8^. In contrast, smaller, soluble IAPP oligomers are believed to exhibit cytotoxic properties^8,14^. Insights from mouse models and human insulinoma suggest that, differing from fibrils, oligomers may also form intracellularly within the β-cells rather than solely extracellularly^8,15,16^. While in vivo the precise impact of the IAPP oligomers on β-cells and hence the reason for their cytotoxicity are not conclusively elucidated, it has been demonstrated that certain oligomer species disrupt the cell membrane^17^. Furthermore, IAPP is a potent activator of the NLRP3 inflammasome, leading to the production of the proinflammatory cytokine IL-1β, which is known to promote insulin resistance and ultimately damage β-cells in the long term^18^.

Aggregation of proteins is a hallmark of many neurodegenerative diseases^19^, but also for systemic protein misfolding diseases. IAPP aggregation may also play a role in the development and progression of T2D^20^. It has been reported that T2D is a risk factor for Alzheimer’s disease (AD) and, vice versa, AD is a risk factor for T2D. One potential explanation for this observation is that IAPP forms fibrils that are very similar to amyloid-beta fibrils^21,22^ suggesting that they potentially can seed each other’s formation and growth^23^.

Hypothetically, IAPP oligomers, once formed, may induce further IAPP aggregation either upon release from β-cells or even inside β-cells even before release into the extracellular space. In order to investigate, whether such a scenario is supported by IAPP oligomer levels in blood of people with T2D compared with healthy controls, we set out to measure IAPP oligomerisation levels in donor blood samples.

We previously developed the sFIDA (surface-based fluorescence intensity distribution analysis) platform method for the quantitative and absolute specific measurement of oligomer species, irrespective of their conformation, morphology, and size, while being insensitive to monomers. This allows quantitation of oligomers even at low femtomolar (fM) concentrations, as demonstrated in human cerebrospinal fluid^24,25^ and complex matrices such as brain homogenates^26^. For this study, we adapted sFIDA for the detection of IAPP aggregates in human plasma EDTA samples. Using the adapted method, oligomers were quantified in samples from individuals clinically diagnosed with T2D, and control samples from metabolically healthy people. Samples from people with T2D were divided into individuals without late complications and those with late complications. Complications comprised nephropathy, retinopathy, and cardiovascular diseases mainly.

The objectives of this study were to elucidate potential differences of IAPP oligomerisation levels in plasma between healthy individuals and people with type 2 diabetes. The acquired insights also aim to resolve whether IAPP oligomers are a cause or a consequence of the disease.

## Methods and materials

### Sampling

Plasma and serum of 29 people with T2D (13 without complications, 16 with complications) and 21 non-diabetic healthy controls were selected from existing samples within the HDZ Biomaterialbank, at the Herz- und Diabeteszentrum Nordrhein-Westfalen in Bad Oeynhausen, Germany. Healthy controls were collected among clinical staff at the hospital after consent in fasting state.

Briefly, blood was centrifuged within two hours after sampling (10 min, 3000 × g) and frozen at − 80 °C until analysis after overnight fasting. The ethics committee of the Ruhr University of Bochum, located in Bad Oeynhausen approved this study (AZ: 2024-1216). All methods were performed in accordance with the relevant guidelines and regulations (Declaration of Helsinki). The diagnosis of diabetes was made based on anamnesis. Data on comorbidities, medication and laboratory values were accessible from regular visits during which sampling occurred, data from healthy donors were freshly accessed at sampling.

### Measurement of total IAPP in human serum

Serum IAPP concentrations were determined by using commercially available ELISA (human Islet Amyloid Polypeptide, cat. No. NBP2-76733, Novus Biologicals, Bio-Techne Europe, UK). Briefly, human serum was diluted 1:4 and measured according to manufacturer’s instructions. The standard used in this kit is the monomeric form of human IAPP 34-70aa (Accession number P10997), against which the detecting antibodies are directed. Specification data are as follows: Sensitivity 37.50 pg/ml, detection range: 62.50–400 pg/ml. Optical density was measured with a micro-plate reader (Tecan, Switzerland) set to 450 nm.

### Measurement of IAPP oligomer in human plasma

**Figure 1:**
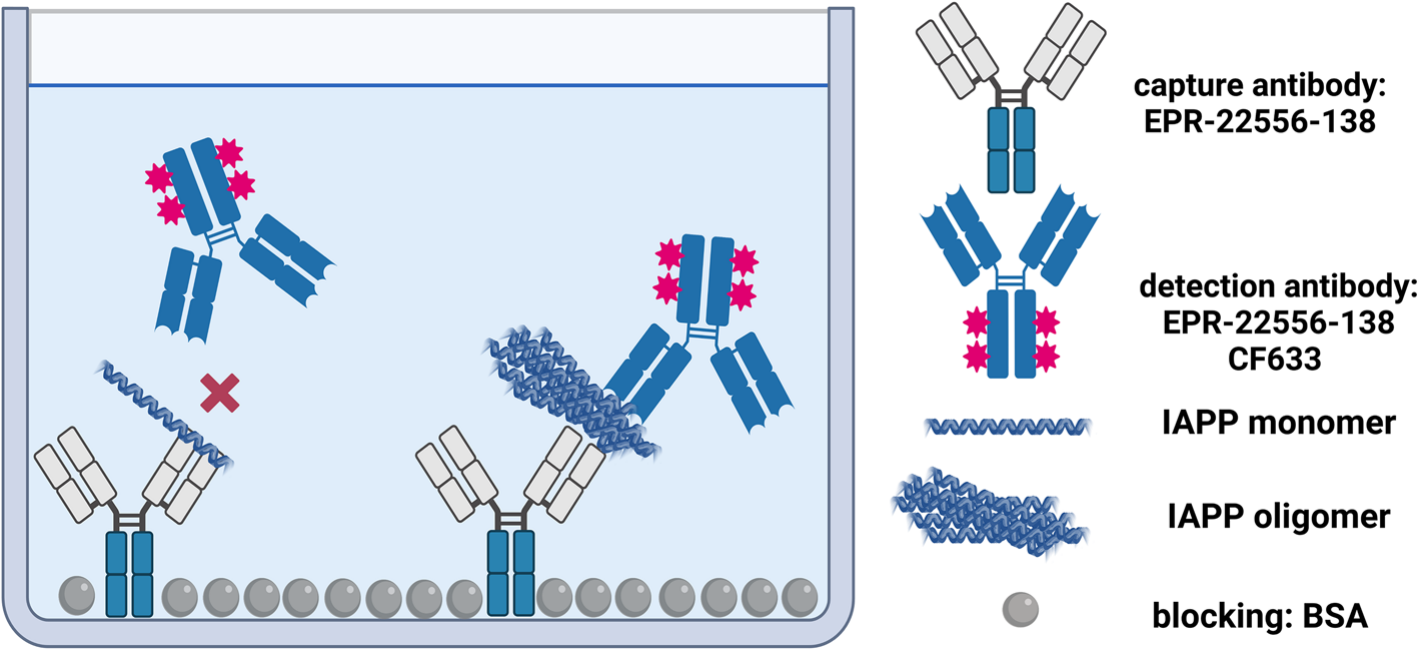
Scheme of surface-based fluorescence intensity distribution analysis. The capture antibody against IAPP (EPR-22556-138) is immobilised on the glass surface of a 384-well plate. IAPP oligomers and monomers can bind the capture antibodies, but only IAPP oligomers can be detected by the fluorescence labelled detection antibody EPR-22556-138 CF633, as the epitope of the monomers is already masked by the capture antibody. Created with BioRender.com.

To determine the concentration of IAPP-aggregates in human plasma EDTA samples we adapted the sFIDA platform technology to IAPP oligomer (see Fig. 1). The general principle of sFIDA was previously described by Blömeke et al.^24^ and Kravchenko et al.^27^.

#### Antibodies and labelling

Thorough selection of antibodies is crucial for a successful assay development. To ensure that only soluble oligomers are detected and monomers are excluded, only antibodies with overlapping or even identical linear epitopes are used in sFIDA. For this purpose, the antibody EPR-22556-138 (ABCAM, Cambridge, UK), which has its epitope at the C-terminal end of the IAPP structure, was used as a capture and fluorescence labelled detection antibody in the present study.

The detection antibody was labelled with CF633 succinimidyl ester (Sigma Aldrich, St. Louis, Missouri, USA) and purified with a polyacrylamide bead suspension (Bio-Gel P-30, Bio-Rad Laboratories, Hercules, USA) according to the manufacturer's protocol. The concentration was determined with UV–Vis (Shimadzu, Kyōto, Japan) and the degree of labelling was determined for quality assurance.

#### Silica-nanoparticles conjugation

We have previously developed silica nanoparticles (SiNaPs) as a standard that can be conjugated with various peptides^24^. For the present work, we conjugate carboxylated SiNaPs (cSiNaPs) with full length IAPP-momomers. Therefore, we solved 1 mg of full-length lyophilised IAPP (Bachem, Bubendorf Switzerland) in 1 ml HFIP and evaporated it in the SpeedVac. Meanwhile, the cSiNaPs were activated with EDC/NHS and added to the evaporated IAPP. After overnight incubation and two washing steps the concentration of the standard was determined by ICP-MS^24^.

#### Surface-based fluorescence intensity distribution analysis method

A pretreated 384 glass bottom microtiterplate (Sensoplate plus, Greiner Bio-One GmbH, Frickenhausen, Germany) and immobilised antibody EPR-22556–138 in a concentration of 1.25 µg/ml in 0.1 M carbonate buffer (Carl Roth, Karlsruhe, Germany) was used for capturing. After an overnight incubation at 4 °C the plate was washed five times with TBS containing 0.05% Tween (TBS-T) and TBS, each. A blocking solution consisting of 3% BSA in TBS with 0.03% ProClin™ 300 (Sigma Aldrich, St. Louis, Missouri, USA) was added to the plate and incubated for 1.5 h at room temperature.

The plate was washed as described above and 20 µl of 1:5 diluted plasma EDTA samples in low cross buffer (LC; Candor Bioscience, Wangen, Germany) were added. In addition, the same volume of an IAPP-SiNaPs calibration dilution was applied, each. For this purpose, the dilution buffer was prepared from plasma EDTA that had previously been diluted 1:5 with LC to ensure comparability with the samples. As negative control 20 µl of 20 pM Aβ-SiNaPs was applied. Subsequently, 20 µl of 0.315 µg/ml EPR-22556-138 CF633 in LC was added to each well after washing five times with TBS and incubated for 1 h at room temperature. To remove excess antibody a final washing step was applied and 80 µl of TBS containing 0.03% ProClin™ was added (Supplementary Fig. 1).

#### Image data acquisition and readout generation

By using a TIRF microscope (Leica Camera AG, Wetzlar, Germany) measurement (excitation: 635 nm, emission filter: 705/22 nm) was carried out with an oil immersion objective in 100 × magnifications as previously described by Kass et al.^26^.

Each sample was applied on 4 wells, from which 25 individual 14-bit grayscale images with a size of 1000 × 1000 pixels were generated. Using the in-house developed sFIDAta software (version 0.61.3), images containing visual artefacts were sorted out. To convert the images into a numerical readout, the number of pixels with intensities above a certain cut-off was counted for each remaining image. Afterwards, outlier detection was conducted at the well level, filtering out all images with a readout of 1.5 interquartile ranges above or below the median of the well. Using the Hodges-Lehman estimator, a single value for each well was calculated. Finally, the average of the four wells is computed to obtain the final readout for a sample. In the following, the generated readout is referred to as the pixel count.

#### Cut-off optimisation

During a fully automated optimisation process, cut-offs are applied to the dilution series at regular intensity intervals of 100. For each cut-off, metrics are computed from the resulting pixel counts to assess the functional quality of the dilution series. The cut-off that, on average, yields the best performance across these metrics, is selected for the analysis, thereby ensuring the most reliable measurement and unbiased analysis of the individual samples. The optimisation metrics are elaborated on below.

#### Metrics

##### Dilution linearity

To achieve optimal distinguishability of pixel counts across various oligomer concentration levels, we employed dilution linearity as an evaluation metric. For this purpose, the dilution linearity of all successive dilution steps, i.e. a concentration ${c}_{i}$ and the further diluted concentration ${c}_{i+1}$ of the SiNaPs series was calculated and subsequently converted into the absolute deviation from the optimum (100%) using Eq. (1). Afterwards, the mean was calculated, normalised using min–max scaling, and transformed into a score using a sigmoid curve (Eq. 2).

1 $$Dilution\,\, linearity=100-abs\left(\frac{{pixel \,\,count}_{{c}_{i+1}}}{\left(\frac{{c}_{i+1}}{{c}_{i}}*{pixel\,\, count}_{{c}_{i}}\right)} \times 100\%\right),$$

2 $${x}{\prime}= \frac{1}{1+{e}^{-\left(x-0.5\right)*8}} \forall 0< x<1.$$

##### Inter-well CV%

Given that the pixel count of each sample is aggregated from 4 replicates, individual replicates with stronger deviations can potentially adversely affect measurement accuracy and reproducibility. To minimize such negative influence, Eq. (3) was used to calculate the coefficient of variation (CV%) between the pixel counts of individual wells at each dilution step within the dilution series. Afterwards, the mean was calculated, normalised using min–max scaling, and transformed into a score using a sigmoid curve (Eq. 2). As the intensity of almost all pixels can exceed very low cut-off values due to background noise, the CV% initially rises with increasing cut-offs. However, this should not be misinterpreted as deterioration, as the actual signal only becomes evident through the reduction of noise associated with an increase in CV%. Therefore, only cut-offs above the cut-off leading to the first local maximum of the CV% are considered.

3 $$CV\%= \frac{\sigma }{\overline{x} } \times 100\%.$$

##### wMSE

As the individual experiments are to be aligned using a weighted linear calibration, the weighted mean square error (wMSE) of such a regression on the SiNaPs dilution series is used as an additional metric. To minimise the residuals even in the low concentration range it was weighted with the reciprocal of the concentration (Eq. 4). Afterwards, the wMSE was normalised using min–max scaling, and transformed into a score using an exponential function (Eq. 5).

4 $$wMSE=\frac{1}{N}\sum_{i=0}^{N}{(\widehat{y}-y)}^{2}\times \frac{1}{y}$$

5 $${x}^{\prime}= \frac{{100}^{x-1}}{99} \forall 0< x<1$$

##### Minimal readout

Another important factor for the evaluation of a cut-off is the minimal pixel count of the dilution series. A high value suggests that the cut-off is set too low, allowing excessive background signal. Conversely, a very low value suggests that the cut-off is set too high, resulting in signal loss and complicating the calibration and analysis process by risking samples without signal. Unlike the previous metrics, no weighting is applied. Instead, a score of one is assigned if the pixel count falls below 1000 while all cut-offs leading to a pixel count below ten are excluded. Further assessment within this range has shown to be ineffective.

#### QScore

Since an increase/decrease of the cut-off does not necessarily lead to improvement/deterioration in all metrics, as some metrics may partly develop inversely, a good compromise between the individual metrics must be found. This is achieved by calculating the average of all metric scores, referred to as the Quality Score (QScore) in the following. Since all metrics included in the average calculation have been normalised, the QScore also falls within the range between zero and one, where one represents the best possible and zero the worst possible value. The cut-off that leads to the highest QScore is subsequently used for the analysis of the individual samples. The results of the optimisation are depicted in the Supplementary Fig. 2.

#### Calibration

To equalise the pixel count level of both experiments by calibration, a linear regression was carried out using the SiNaPs dilution series between 0 and 2 pM. The individual dilution points were weighted with the reciprocal of their concentration to minimise residuals even in the low concentration range. Both regressions delivered a very high coefficient of determination of *0.998* and *0.990*. When applying the regression model, the intercept was subtracted from the prediction.

### Oligomerisation level

Given that IAPP monomer is co-secreted with insulin in β-cells, whose amount diminishes at differing rates and extents depending on the progression of diabetes, the quantity of available monomer for aggregation consequently fluctuates in accordance with the unique course of the disease. For this reason, it does not seem reasonable to directly compare the oligomer titres of different individuals without considering the total amount of monomers available for aggregation, due to the fluctuation of total IAPP during disease progression. Therefore, the IAPP oligomer concentration, measured by sFIDA (2.3) was normalised by the total IAPP concentration (2.2). The result of this normalisation will be referred to as the oligomerisation level in the subsequent text.

### Statistics

To select the appropriate statistical test for each investigation, various tests for normal distribution were conducted, namely the Shapiro–Wilk test, Kolmogorov–Smirnov test, and D’Agostino’s K-squared test. In the case of not normal distribution data, non-parametric tests are employed for data analyses. Statistical analysis as well as the calibration were performed and illustrated using python 3.9.7 (Python software foundation, Wilmington, USA; packages: pandas 1.3.4, numpy 1.26.3, scipy 1.7.1, sklearn 1.0.2, seaborn 0.11.2).

### Ethics approval and consent to participate

All local institutional review boards and ethical committees approved the study protocol. All participants gave informed consent before inclusion in the study.

## Results

The aim of the present study was to investigate IAPP oligomerisation levels in human plasma samples of healthy controls and of people with type 2 diabetes.

### Descriptive analysis of individual groups

Samples were selected from HDZ NRW Biomaterialbank comprising individuals with T2D with or without late complications (mainly nephropathy, defined by presence of albuminuria), and healthy controls sampled among employees of the HDZ NRW. Basic characteristics are given in Table 1.

**Table 1:** Basic characteristics of analysed cohort comprising clinical data, therapy and co-morbidities.

Parameter	Healthy control	T2D without complications	T2D with complications
Number	21	13	16
Age [yrs]	48.9 ± 7.2	56.9 ± 9.9	62.9 ± 18.2
BMI [kg/m^2^]	27.1 ± 4.2	28.2 ± 3.6	29.8 ± 4.0
Diabetes duration [yrs]	0.0 ± 0.0	5.2 ± 4.4	16.1 ± 12.5
HbA1c [%]	5.3 ± 0.2	7.9 ± 1.2	8.4 ± 1.0
C-peptide [pmol/l]	1067 ± 557	1275 ± 439	1100 ± 832
HDL-cholesterol [mg/dl]	53.0 ± 11.1	45.1 ± 8.8	44.3 ± 9.3
LDL-cholesterol [mg/dl]	142.4 ± 23.8	134.2 ± 48.8	93.7 ± 27.4
GFR [ml/min]	–	82.2 ± 13.7	78.3 ± 18.8
Urine albumine [mg/l]	–	16.0 ± 18.4	75.9 ± 46.8
Blood pressure systolic [mm Hg]	–	128.0 ± 7.0	144.0 ± 14.0
Blood pressure diastolic [mm Hg]	–	77.0 ± 8.0	84.0 ± 11.0
IAPP [pg/ml]	3701 ± 4771	1165 ± 647	4906 ± 6457
Insulin in mU/l (includes insulin therapy)	15.2 ± 10.7	17.6 ± 27.5	33.9 ± 21.5
SPISE-INDEX	5.9 ± 1.5	5.1 ± 1.3	4.7 ± 1.0
Therapy			
Insulin (U/day/kg BW)			0.538 ± 0.219
Metformin [N]		10	6
SGLT2 [N]		3	4
DPPIV [N]		6	3
Statin [N]		3	10
Omega-3-fatty acids [N]		4	4
Ezetimibe [N]		1	0
Fibrate [N]		0	2
Co-morbidities			
Hypertension [N]		6	14
PAD [N]		0	4
CVD [N]		0	5
CAD [N]		0	8
DFU [N]		0	4

To select appropriate statistical tests, an assessment was made to determine if the data aligned with a normal distribution. Given the deviation from normal distribution (Supplementary Table 1), subsequent statistical analyses were conducted using non-parametric tests.

To exclude the influence of demographic factors such as age or sex on the data, statistical investigations were conducted. Spearman analyses within each diagnostic group demonstrated that there is no significant correlation between age and oligomerisation levels in any group (Supplementary Table 2). Additionally, it has been shown that there is no significant sex-specific difference in the oligomerisation level for any diagnostic group (Supplementary Table 3).

### People with T2D without complications showed significantly higher IAPP oligomerisation levels compared to controls

**Figure 2:**
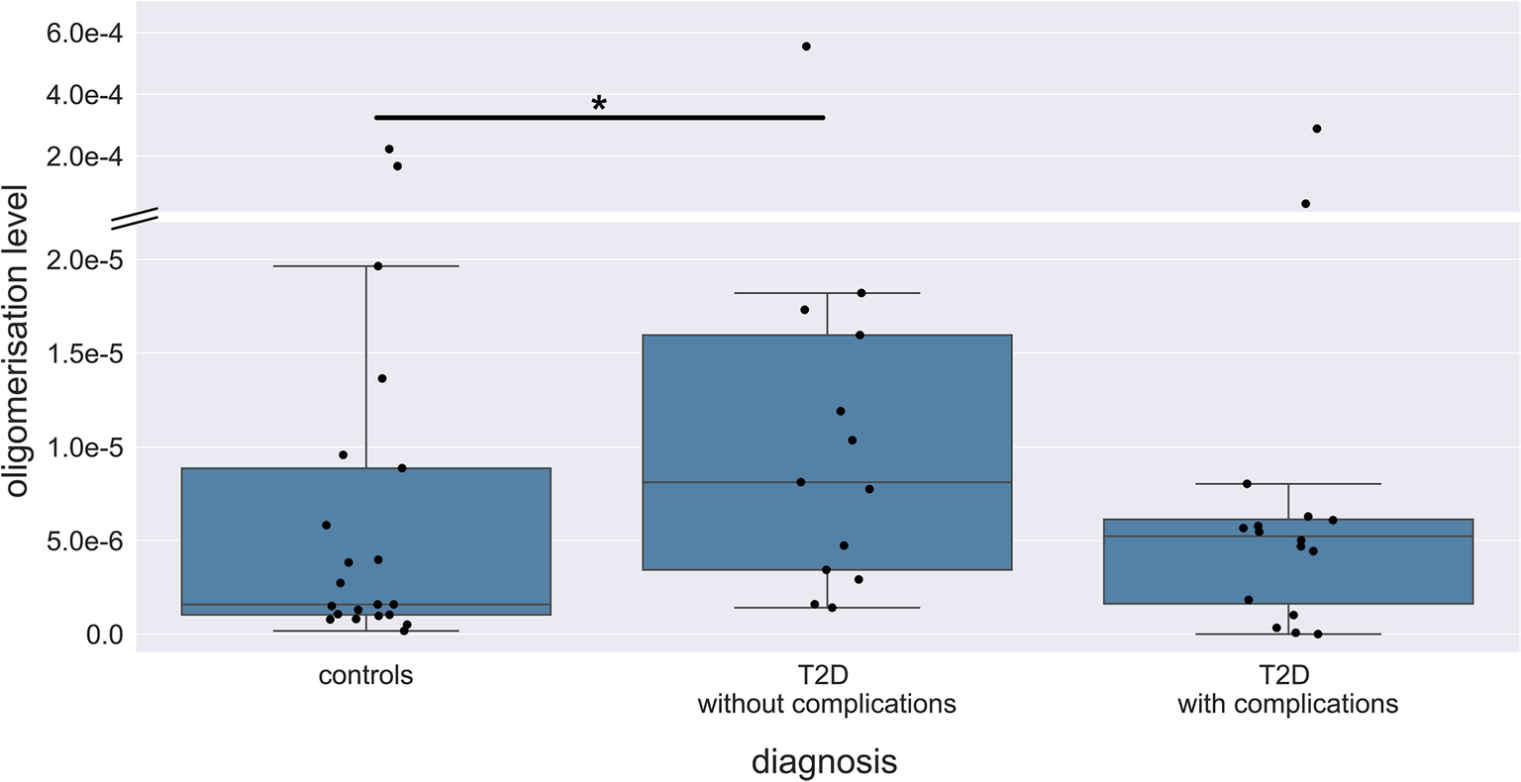
Oligomerisation levels based on diabetes subgroups. Oligomerisation levels of T2D group without complications are significantly elevated compared to the controls (*P* value: 0.037). ● indicates single individuals. – indicates the median. A two-sided Mann–Whitney *U* Test (confidence interval: 0.05) was carried out to investigate differences between the groups. Significant differences were labelled with *. Please, note the two-part y-axis, which removes free space.

First, we investigated the levels of IAPP oligomerisation within the different groups using two-sided Mann–Whitney *U* test. For people with T2D without complications, a statistically significant elevation in the oligomerisation level was observed compared to the control group (*P* value: 0.037, Fig. 2) with an effect size, measured as the percentage increase of the median, being 412% higher than the control group. Although the median of the T2D group with complications is elevated compared to the control group (by 231%), there is no significant difference to the other groups due to heterogenous value distribution. A ROC curve was generated to enable a further evaluation of the diagnostic quality (Supplementary Fig. 3).

### Positive correlation between oligomerisation level and disease duration in people with T2D without complications

**Figure 3:**
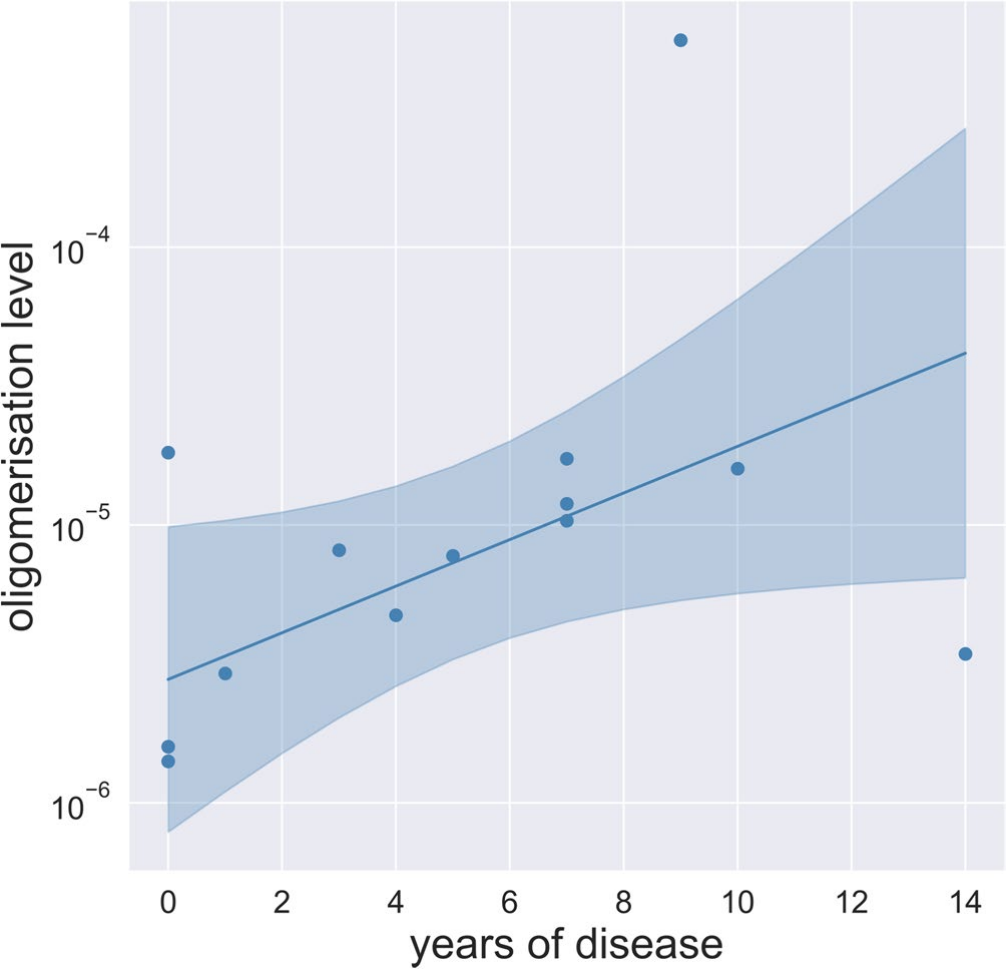
Relationship between oligomerisation level and disease duration in people with T2D without complications. In the first 10 years the oligomerisation level of T2D group without complications correlates strongly positive with the duration of the disease (Spearman* r* value: 0.617, *P *value: 0.033). The line represents a fitted Huber regression. The surrounding marked area represents the confidence interval. Data for the entirety of the T2D subgroups are shown in Supplementary Fig. 4.

In addition to inter-group comparisons of oligomerisation levels, an investigation into the temporal dynamics of oligomerisation level increase was undertaken. Specifically, an assessment was conducted within each cohort to ascertain whether a correlation exists between the oligomerisation level and the duration of diabetes. For this purpose, the two-sided Spearman correlation test was performed. For the entirety of the T2D subgroups, no correlation is observed between the years of disease and oligomerisation level (r value: − 0.122, *P* value: 0.528). However, when the analysis is restricted to the first 10 years, a moderately strong positive correlation emerges, although it is not statistically significant (r value: 0.392, *P* value: 0.097). A further refinement into the T2D subgroups shows a strong, positive, and significant correlation for the T2D group without complications (r value: 0.617, *P* value: 0.033). Conversely, the T2D group with complications does not exhibit any correlation within the same time frame (r value: − 0.054, *P* value: 0.908). Subsequently, a Huber regression, which is robust against outliers, was fitted to the oligomerisation levels and diabetes duration of T2D group without complications. The median value of the control group closely aligns with the intercept of the Huber regression displayed in Fig. 3 (Median = 1.59e–6, Intercept = 2.78e–6). However, due to the limited data set of T2D without complications, especially with a disease duration of more than seven years it is not possible to reliably estimate if and when the oligomerisation level will peak. Further correlations are listed in Supplementary Tables 4–7.

## Discussion

The main focus of this research was to demonstrate a relation between the stage of the disease without comorbidities and complications and insulin applications—and IAPP oligomerisation levels. To create the oligomerisation level, we quantified concentrations of IAPP oligomers in plasma using sFIDA and normalised them to the total IAPP concentration in serum to account for the individually very different total IAPP levels. People with T2D without complications presented with the highest IAPP oligomerisation levels whereas healthy controls showed the lowest levels as expected. In T2D group without complications, oligomerisation levels were significantly elevated compared to the control group. In T2D group with complications, mainly albuminuria with preserved GFR, the median oligomerisation level is also elevated compared to the control group; however, statistical significance could not be established. These observations support that elevated IAPP aggregation exists in people with T2D. Within the first 10 years of the disease, oligomerisation level of T2D group was in correlation to the duration of the disease as long as no confounding conditions, like comorbidities and/or insulin applications are present. Although this cannot prove that IAPP oligomerisation is the driving factor of type 2 diabetes development and progression, one would expect that the IAPP oligomerisation level is increasing with disease duration. This might open the opportunity to determine the real disease onset, long before the disease becomes diagnosed. Next to the lower oligomerisation levels in T2D group with complications, there is also no correlation between oligomerisation levels and the duration of the disease.

There are various plasma components that are supposed to have a stabilising effect on IAPP oligomers or inhibit their aggregation. Camargo et al.^28^ show that sucrose (glucose, fructose) has a stabilising effect on IAPP oligomers. In contrast, we were unable to demonstrate that HbA1c influences the oligomerisation level. This could be due to the possibility that only short-term effects exist, which are not detectable by long-term glycaemic markers. Insulin, on the other hand, is a potential inhibitor for the aggregation of IAPP oligomers^29,30^. In accordance with this, we observed an inverse correlation between insulin and the oligomerisation levels of all samples, which was particularly pronounced and significant in the control group (Supplementary Table 8). However, no such correlation was found for people with T2D. The absence of this correlation supports the hypothesis that additional factors must be involved in T2D, or that the binding of insulin to IAPP is influenced. Whether insulin therapy might destabilise IAPP oligomers and result in their partial reduction remains an open question^31^. IAPP fibrils and plaques form not only in the pancreas of persons with T2D but also in the brain, which is not the case for healthy people^32^. Furthermore, IAPP can form fibrils that are very similar to amyloid-beta fibrils^21,22^ suggesting that T2D and AD potentially can seed each other’s formation and growth^23^. Taken together with our results, which demonstrate that the aggregation of IAPP into soluble oligomers is increased in T2D and progresses over the course of the disease, this may be one potential explanation, why T2D is a risk factor for Alzheimer’s disease^33,34^.

Based on our results, it is neither possible to use the oligomerisation level to assess the severity of the disease, nor to determine whether IAPP oligomerisation can lead to the occurrence of complications. Due to the lack of data from a T2D group without complications with high diabetes duration, it is currently not possible to make a reliable statement about the duration at which the oligomer progression reaches its peak and how it behaves thereafter. We have chosen different matrices for the measurement of total IAPP and IAPP oligomers because serum is recommended for metabolomic measurements^35^ and the commercially available ELISA as well, but results in a lower signal-to-noise ratio and consequently lower accuracy in sFIDA measurements compared to plasma, making plasma the better choice for this assay. As the composition of serum and plasma differs, this might influence the results. Moreover, it should be considered that IAPP in plasma can potentially bind to other plasma components such as apolipoproteins^36^ and beta-amyloid^37^. Even if this does not lead to false positive signals in the sFIDA assay, it should be considered when interpreting the results.

To clarify the open questions, a longitudinal observation of a larger group of people with T2D would be useful. The further development of the level of oligomerisation could be clarified, and if complications develop in some people over time, conclusions could be drawn from the previously recorded oligomerisation levels. Because IAPP oligomerisation levels were increased in T2D patients without complications in comparison to healthy controls, determination of IAPP oligomers in clinically healthy but obese people might become a tool to identify people at risk to develop T2D. Furthermore, the correlation between oligomerisation levels and the duration of diabetes, might open the possibility to determine the real diabetes time, before overt hyperglycaemia is diagnosed. This, of course, requires many more studies to investigate this potential. IAPP oligomers have been widely detected in pancreatic tissue and oligomerisation is regarded as main contributor to loss of ß-cell function and number. Whether plasma concentrations correlate to pancreatic levels remains elusive as pancreatic biopsies have not been analysed in parallel, and therefore require further investigation.

## Conclusion

Taken together we have shown for the first time that the sFIDA technology works in blood plasma from people with type 2 diabetes to determine IAPP oligomer concentrations down to the fM range. Additional work has to be done to prove the results in a higher number of individuals in different disease stages, according to comorbidities and background therapy. Comparative tissue analysis is warranted to rule out issues on secretion and serum stability of IAPP. Highly sensitive analysis methods for systemic detection of organ-specific misfolded proteins may develop as a future analysis method to identify early organ damage.

### Supplementary Information

Supplementary Information.

## Data Availability

The authors confirm that the data supporting the findings of this study are available within the article and its supplementary materials.
