# Supplementary material for: IAPP - oligomerisation levels in plasma of people with type 2 diabetes
Source: Sci Rep. 2024 Aug 22;14:19556. doi: 10.1038/s41598-024-70255-3 (PMC11341561; doi:10.1038/s41598-024-70255-3)
Supplement: Supplementary file 1 — Supplementary Information. [file 41598_2024_70255_MOESM1_ESM.pdf]

IAPP - oligomerisation levels in plasma of people with type 2 diabetes

Authors: Fabian Rehn[a,1,2,3], Victoria Kraemer-Schulien[a,2], Tuyen Bujnicki[2], Oliver Bannach[2,1,3], Diethelm Tschoepe[4,5], Bernd Stratmann[b,4*], Dieter Willbold[b,2,1,3*]

a: All authors contributed equally. The order was set randomly.

b: Lead Contact, shared last authorship

[1]Institut für Physikalische Biologie, Heinrich-Heine-Universität Düsseldorf, Universitätsstr. 1, 40225 Düsseldorf, Germany.

[2]Institute of Biological Information Processing (Structural Biochemistry: IBI-7), Forschungszentrum Jülich GmbH, Wilhelm-Johnen-Straße, 52428 Jülich, Germany.

[3]attyloid GmbH, Merowingerplatz 1A, 40225 Düsseldorf, Germany.

[4]Herz- und Diabeteszentrum Nordrhein-Westfalen, Universitätsklinik der Ruhr-Universität Bochum, Medizinische Fakultät OWL (Universität Bielefeld), Georgstr. 11, D-32545 Bad Oeynhausen, Germany

[5]Stiftung DHG (Diabetes I Herz I Gefäße) in der Deutschen Diabetes Stiftung c/o Deutsches Diabetes-Zentrum (DDZ), Auf´m Hennekamp 65, 40225 Düsseldorf, Germany

*Corresponding Authors:

Prof. Dr. Dieter Willbold, Forschungszentrum Jülich GmbH, Institute of Biological Information Processing (IBI-7), Forschungszentrum Jülich, Wilhelm-Johnen-Straße, 52428 Jülich, Germany

Tel.: +49 2461 61 2100; E-Mail: d.willbold@fz-juelich.de

PD Dr. rer. nat. Bernd Stratmann, Herz- und Diabeteszentrum Nordrhein-Westfalen, Universitätsklinik der Ruhr-Universität Bochum, Medizinische Fakultät OWL (Universität Bielefeld), Georgstr. 11, D-32545 Bad Oeynhausen, Germany

Tel. 05731 973768, E-Mail: bstratmann@hdz-nrw.de

Supplement

Sup. Table 1: Test for normal distribution

Test	*P* value
Shapiro-Wilk	*1.306e-13*
Kolmogorov-Smirnov	*4.393e-12*
D'Agostino's K-squared	*7.980e-18*

To select suitable statistical methods, the oligomerisation level was analysed using various tests for normal distribution. None of the tests indicated a normal distribution.

Sup. Table 2: Confounding factor: Age

Diagnosis group	r value	*P* value
control	-0.207	0.368
T2D without complications	-0.069	0.823
T2D with complications	-0.142	0.600

Using a two-sided Spearman correlation test, it was investigated whether there is a correlation between oligomerisation level and age in any of the diagnostic groups. No significant correlation was found in any of the groups.

Sup. Table 3: Confounding factor: Gender

Diagnostic group	*P* value
control	0.916
T2D without complications	0.231
T2D with complications	0.417

Using a two-sided Mann-Whitney U test, it was investigated whether there are significant differences in oligomerisation levels depending on gender within the diagnostic groups. No significant differences were found in any of the groups.

Sup. Table 4: Correlation of IAPP with disease years

T2D group	IAPP Type	r value	*P* value
Without complications	Oligomerisation level	0.439	0.133
Without complications	Oligomer concentration	0.433	0.139
Without complications	Total IAPP concentration	-0.189	0.537
With complications	Oligomerisation level	-0.394	0.132
With complications	Oligomer concentration	-0.619	0.011
With complications	Total IAPP concentration	-0.389	0.137

Using a two-sided Spearman correlation test, the correlation between various IAPP types and the duration of the disease in years was investigated.

Sup. Table 5: Correlation of IAPP with disease years during first 10 years

T2D group	IAPP Type	r value	*P* value
Without complications	Oligomerisation level	0.617	0.033
Without complications	Oligomer concentration	0.688	0.013
Without complications	Total IAPP concentration	-0.298	0.347
With complications	Oligomerisation level	-0.054	0.908
With complications	Oligomer concentration	0.162	0.728
With complications	Total IAPP concentration	-0.391	0.386

Using a two-sided Spearman correlation test, the correlation between various IAPP types and the duration of the disease in years was investigated. Data is limited to a maximum disease duration of 10 years.

Sup. Table 6: Correlation of IAPP with HbA1c [in % (4,3-6,1)]

T2D group	IAPP Type	r value	*P* value
Without complications	Oligomerisation level	0.320	0.287
Without complications	Oligomer concentration	0.163	0.596
Without complications	Total IAPP concentration	-0.661	0.014
With complications	Oligomerisation level	0.279	0.295
With complications	Oligomer concentration	-0.007	0.978
With complications	Total IAPP concentration	-0.231	0.389

Using a two-sided Spearman correlation test, the correlation between various IAPP types and HbA1c in serum was investigated.

Sup. Table 7: Correlation of IAPP with HbA1c [in % (4,3-6,1)] during first 10 years

T2D group	IAPP Type	r value	*P* value
Without complications	Oligomerisation level	0.340	0.280
Without complications	Oligomer concentration	0.196	0.541
Without complications	Total IAPP concentration	-0.690	0.013
With complications	Oligomerisation level	-0.143	0.760
With complications	Oligomer concentration	-0.571	0.180
With complications	Total IAPP concentration	-0.198	0.670

Using a two-sided Spearman correlation test, the correlation between various IAPP types and the HbA1c in serum was investigated. Data is limited to a maximum disease duration of 10 years.

Sup. Table 8: Correlation of IAPP oligomerisation levels with Insulin mU/l in serum

Group	r value	*P* value
All Samples	-0.277	0.052
Control	-0.517	0.017
T2D (both subgroups)	-0.249	0.193

Using a two-sided Spearman correlation test, the correlation between IAPP oligomerisation level and insulin in serum was investigated.

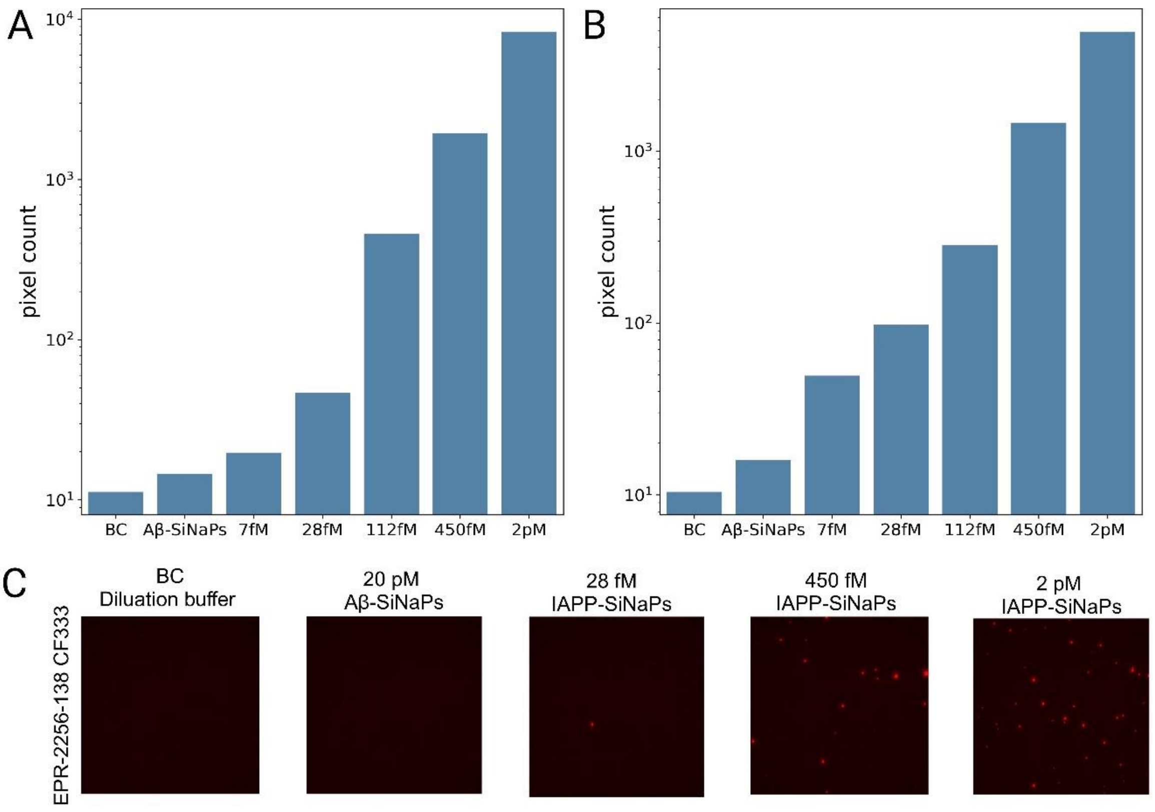

Sup. Figure 1: Assay Controls

Figure A) and B) shows assay controls for the two individual experiments. For both experiments, the BC provides the lowest signals. Aβ-SiNaPs lead to a slight increase in signal in both cases, however, it should be noted that non-physiologically high concentrations (20 pM) were used here, which means that no influence on the results is to be expected at physiological concentrations. It is also shown that the signal increases considerably with increasing concentrations (7 fM – 2 pM) of IAPP SiNaPs. Figure C) show the corresponding TIFFs Images.

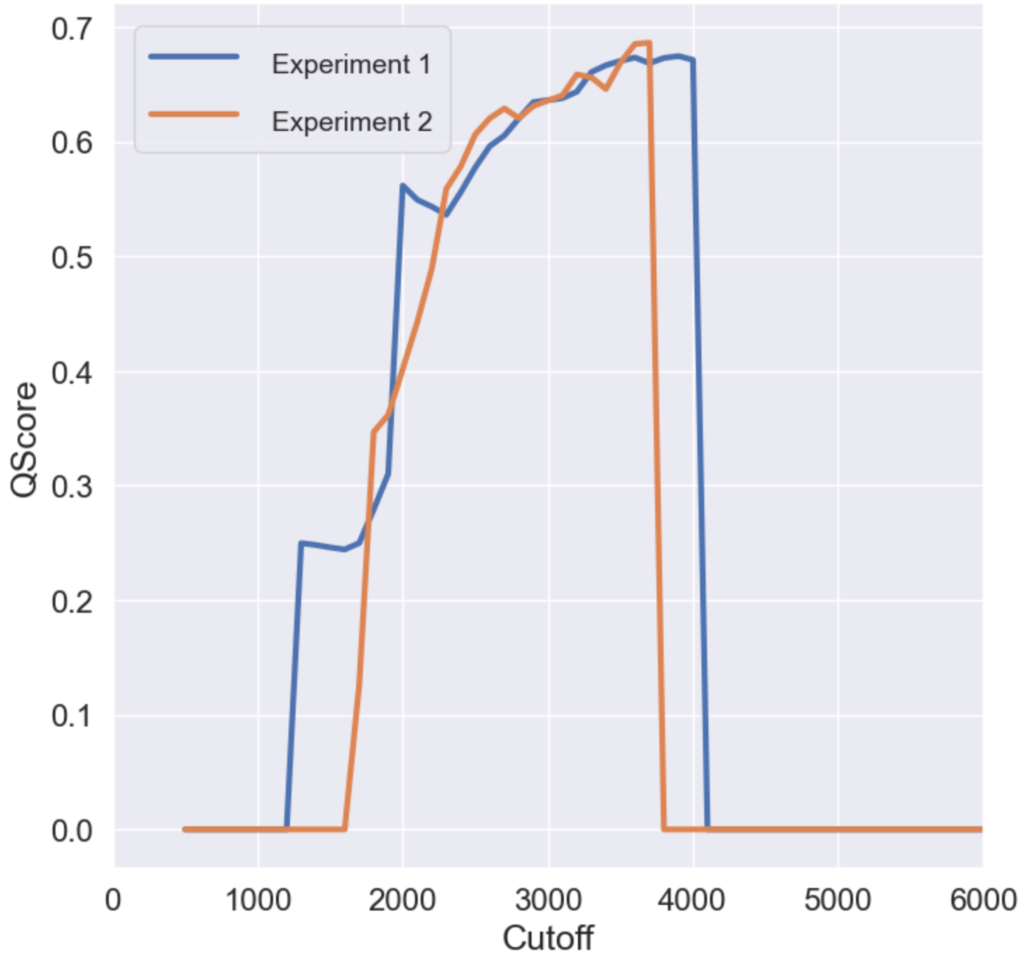

Sup. Figure 2: Cut-off optimisation results

QScores of the individual experiments in relation to the applied cut-off. It shows that the QScore develops very similarly in both experiments. The maximum QScore of 0.675 and 0.686 is reached at a cut-off of 3900 and 3700 respectively.

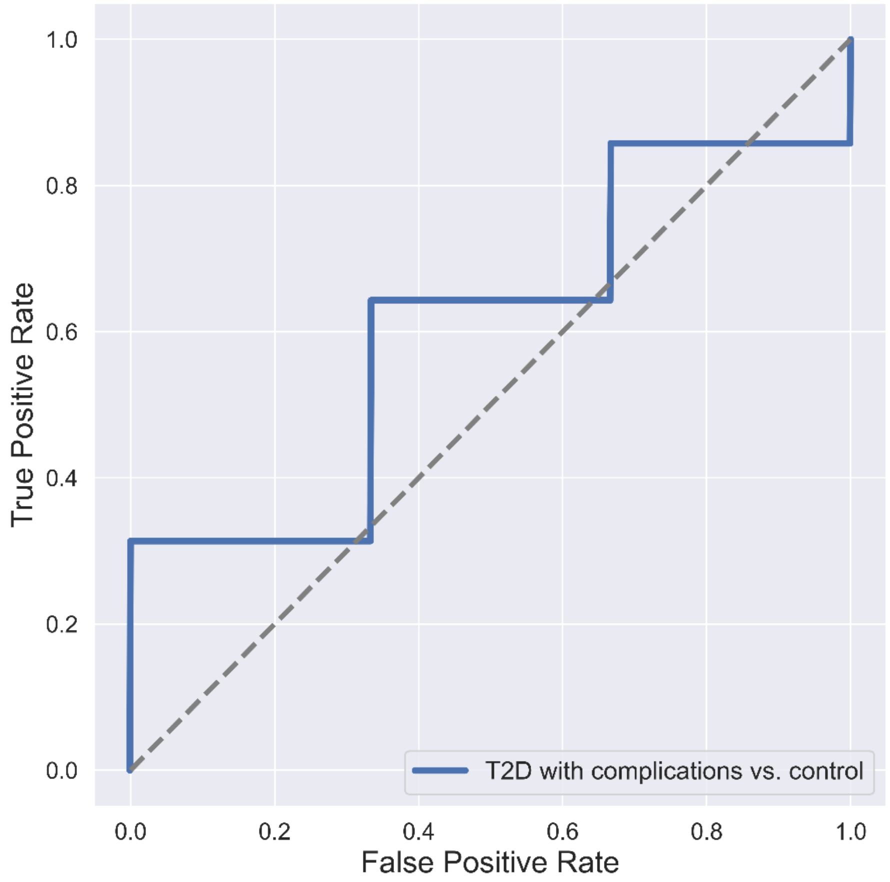

Sup. Figure 3: ROC-Curves

ROC analysis was carried out to assess the ability to classify individuals based on the oligomerisation level. For this purpose, a randomised and stratified split into training and test set was carried out. ROC curves were then created using a logistic regression. Due to the small group size, the result depends heavily on the split. Therefore, the procedure was repeated a thousand times with different random seeds to determine the mean ROC curve and the mean AUC (0.61).

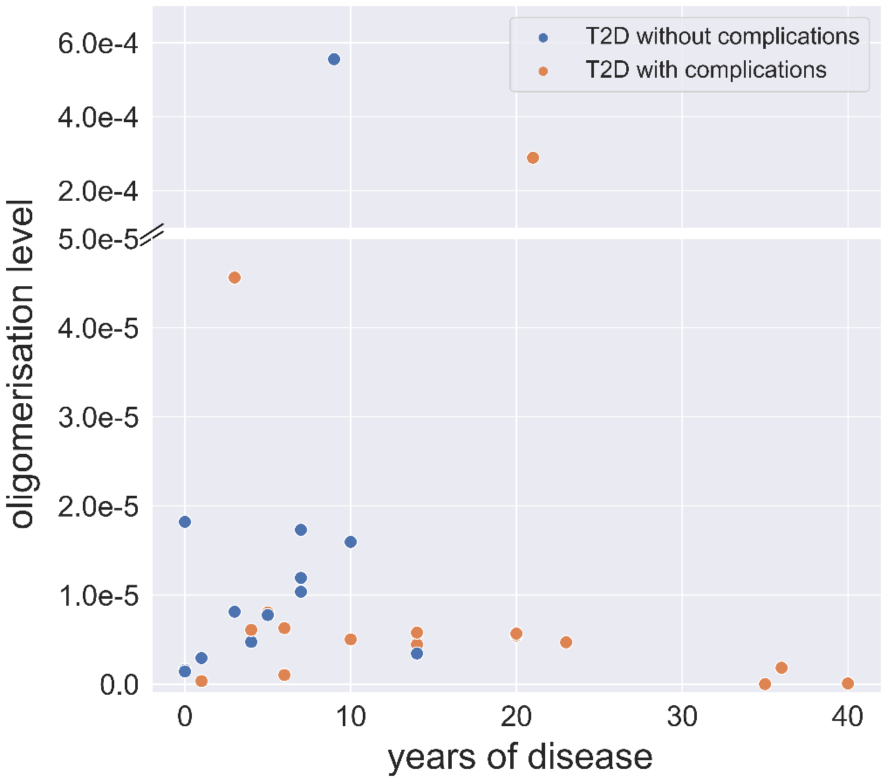

Sup. Figure 4: T2D oligomerisation level in relation to the years of disease

Relation between oligomerisation level and years of disease of both T2D subgroups. Please, note the two-part y-axis, which removes free space.

References

1 Polonsky, K. Dynamics of insulin secretion in obesity and diabetes. *International journal of obesity* **24**, S29-S31 (2000).

2 Butler, A. E. *et al.* β-cell deficit and increased β-cell apoptosis in humans with type 2 diabetes. *Diabetes* **52**, 102-110 (2003).

3 Meir, J. *et al.* The vascular complications of diabetes: a review of their management, pathogenesis, and prevention. *Expert Review of Endocrinology & Metabolism* **19**, 11-20 (2024).

4 Orozco, L. J. *et al.* Exercise or exercise and diet for preventing type 2 diabetes mellitus. *Cochrane database of systematic reviews* (2008).

5 Sladek, R. *et al.* A genome-wide association study identifies novel risk loci for type 2 diabetes. *Nature* **445**, 881-885 (2007).

6 Chatterjee, S., Khunti, K. & Davies, M. J. Type 2 diabetes. *The lancet* **389**, 2239-2251 (2017).

7 Abdul Basith Khan, M. *et al.* Epidemiology of type 2 diabetes—global burden of disease and forecasted trends. *Journal of epidemiology and global health* **10**, 107-111 (2020).

8 Haataja, L., Gurlo, T., Huang, C. J. & Butler, P. C. Islet amyloid in type 2 diabetes, and the toxic oligomer hypothesis. *Endocrine reviews* **29**, 303-316 (2008).

9 DiMeglio, L. A., Evans-Molina, C. & Oram, R. A. Type 1 diabetes. *The Lancet* **391**, 2449-2462 (2018).

10 Kahn, S. E. *et al.* Evidence of cosecretion of islet amyloid polypeptide and insulin by β-cells. *Diabetes* **39**, 634-638 (1990).

11 Mulder, H., Ahren, B., Stridsberg, M. & Sundler, F. Non-parallelism of islet amyloid polypeptide (amylin) and insulin gene expression in rat islets following dexamethasone treatment. *Diabetologia* **38**, 395-402 (1995).

12 Westermark, P., Engström, U., Johnson, K. H., Westermark, G. T. & Betsholtz, C. Islet amyloid polypeptide: pinpointing amino acid residues linked to amyloid fibril formation. *Proceedings of the National Academy of Sciences* **87**, 5036-5040 (1990).

13 Kahn, S. E., Andrikopoulos, S. & Verchere, C. B. Islet amyloid: a long-recognized but underappreciated pathological feature of type 2 diabetes. *Diabetes* **48**, 241-253 (1999).

14 Janson, J., Ashley, R. H., Harrison, D., McIntyre, S. & Butler, P. C. The mechanism of islet amyloid polypeptide toxicity is membrane disruption by intermediate-sized toxic amyloid particles. *Diabetes* **48**, 491-498 (1999).

15 Gurlo, T. *et al.* Evidence for proteotoxicity in β cells in type 2 diabetes: toxic islet amyloid polypeptide oligomers form intracellularly in the secretory pathway. *The American journal of pathology* **176**, 861-869 (2010).

16 Lin, C.-Y. *et al.* Toxic human islet amyloid polypeptide (h-IAPP) oligomers are intracellular, and vaccination to induce anti-toxic oligomer antibodies does not prevent h-IAPP–induced β-cell apoptosis in h-IAPP transgenic mice. *Diabetes* **56**, 1324-1332 (2007).

17 Kanatsuka, A., Kou, S. & Makino, H. IAPP/amylin and β-cell failure: Implication of the risk factors of type 2 diabetes. *Diabetology international* **9**, 143-157 (2018).

18 Masters, S. L. *et al.* Activation of the NLRP3 inflammasome by islet amyloid polypeptide provides a mechanism for enhanced IL-1β in type 2 diabetes. *Nature immunology* **11**, 897-904 (2010).

19 Willbold, D., Strodel, B., Schröder, G. F., Hoyer, W. & Heise, H. Amyloid-type protein aggregation and prion-like properties of amyloids. *Chemical reviews* **121**, 8285-8307 (2021).

20 Moreno-Gonzalez, I. *et al.* Molecular interaction between type 2 diabetes and Alzheimer’s disease through cross-seeding of protein misfolding. *Molecular psychiatry* **22**, 1327-1334 (2017).

21 Röder, C. *et al.* Cryo-EM structure of islet amyloid polypeptide fibrils reveals similarities with amyloid-β fibrils. *Nature structural & molecular biology* **27**, 660-667 (2020).

22 Gremer, L. *et al.* Fibril structure of amyloid-β (1–42) by cryo–electron microscopy. *Science* **358**, 116-119 (2017).

23 Hu, R., Zhang, M., Chen, H., Jiang, B. & Zheng, J. Cross-seeding interaction between β-amyloid and human islet amyloid polypeptide. *ACS chemical neuroscience* **6**, 1759-1768 (2015).

24 Blömeke, L. *et al.* Quantitative detection of α-Synuclein and Tau oligomers and other aggregates by digital single particle counting. *npj Parkinson's Disease* **8**, 68 (2022).

25 Blömeke, L. *et al.* Aβ oligomers peak in early stages of Alzheimer's disease preceding tau pathology. *Alzheimer's & Dementia: Diagnosis, Assessment & Disease Monitoring* **16**, e12589 (2024).

26 Kass, B. *et al.* Aβ oligomer concentration in mouse and human brain and its drug-induced reduction ex vivo. *Cell Reports Medicine* **3** (2022).

27 Kravchenko, K. *et al.* Analysis of anticoagulants for blood-based quantitation of amyloid β oligomers in the sFIDA assay. *Biological chemistry* **398**, 465-475 (2017).
28 Camargo, D. C. R. *et al.* hIAPP forms toxic oligomers in plasma. *Chemical Communications* **54**, 5426-5429 (2018).
29 Larson, J. L. & Miranker, A. D. The mechanism of insulin action on islet amyloid polypeptide fiber formation. *Journal of molecular biology* **335**, 221-231 (2004).
30 Baram, M., Gilead, S., Gazit, E. & Miller, Y. Mechanistic perspective and functional activity of insulin in amylin aggregation. *Chemical science* **9**, 4244-4252 (2018).
31 Gilead, S., Wolfenson, H. & Gazit, E. Molecular mapping of the recognition interface between the islet amyloid polypeptide and insulin. *Angewandte Chemie* **118**, 6626-6630 (2006).
32 Jackson, K. *et al.* Amylin deposition in the brain: a second amyloid in Alzheimer disease? *Annals of neurology* **74**, 517-526 (2013).
33 Amidei, C. B. *et al.* Association between age at diabetes onset and subsequent risk of dementia. *Jama* **325**, 1640-1649 (2021).
34 Athanasaki, A. *et al.* Type 2 diabetes mellitus as a risk factor for Alzheimer's disease: Review and meta-analysis. *Biomedicines* **10**, 778 (2022).
35 Liu, L. *et al.* Differences in metabolite profile between blood plasma and serum. *Analytical biochemistry* **406**, 105-112 (2010).
36 Ly, H. *et al.* Brain microvascular injury and white matter disease provoked by diabetes-associated hyperamylinemia. *Annals of neurology* **82**, 208-222 (2017).
37 Kotiya, D. *et al.* Rapid, scalable assay of amylin-β amyloid co-aggregation in brain tissue and blood. *Journal of Biological Chemistry* **299** (2023).
